# Prevalence and outcome of Ventilator-associated pneumonia (VAP) in living donor liver transplant (LDLT) patients in a developing country

**DOI:** 10.12669/pjms.42.4.15082

**Published:** 2026-04

**Authors:** Saleem Sharieff, Iqbal Hussain, Farrah Tariq, Sadia Ghulam Mustafa, Faizan Rathoor, Arslan Saleem Chughtai

**Affiliations:** 1 Saleem Sharieff, MD, FCPS, FRCPC (Internal Medicine & Critical Care Medicine) Consultant Intensivist, Pakistan Kidney and Liver Institute and Research Centre (PKLI-RC), Lahore, Pakistan; 2 Iqbal Hussain, MD, DABIM, DABCCM. Pakistan Kidney and Liver Institute and Research Centre (PKLI-RC), Lahore, Pakistan; 3 Farrah Tariq, MBBS, FCPS, MRCP Fellow Critical Care Medicine, Pakistan Kidney and Liver Institute and Research Centre (PKLI-RC), Lahore, Pakistan; 4 Sadia Ghulam Mustafa, MBBS, MPH, FISqua Clinical Governance Officer, Medical Services, Pakistan Kidney and Liver Institute and Research Centre (PKLI-RC), Lahore, Pakistan; 5 Faizan Rathoor, MBBS, FCPS Senior Registrar ICU, Pakistan Kidney and Liver Institute and Research Centre (PKLI-RC), Lahore, Pakistan; 6 Arslan Saleem Chughtai, M. Phil (Stats) Biostatistician, Pakistan Kidney and Liver Institute and Research Centre (PKLI-RC), Lahore, Pakistan

**Keywords:** ICU length of stay, Liver transplant, Mortality, Ventilator-associated pneumonia

## Abstract

**Background and Objectives:**

Patients using mechanical ventilation for more than 48 hours may develop ventilator-associated pneumonia (VAP). There is limited data on the prevalence of VAP following liver transplantation from developing countries, especially in living donor liver transplant (LDLT) patients. Our study’s goals were to ascertain the prevalence, risk factors, and outcomes of VAP in patients who have undergone LDLT.

**Methodology:**

This retrospective study reviewed 324 patients who underwent LDLT at the Pakistan Kidney and Liver Institute and Research Centre (PKLI-RC) between January 1^st^, 2022 to December 31^st^, 2023. VAP was identified based on microbiological and clinical criteria. Clinical characteristics, demographics, and mortality outcomes were recorded.

**Results:**

Among 324 liver transplant patients, 253 (78.1%) were male and 71 (21.9%) were female. Hepatitis C virus (HCV; 58.95%) was the most common cause of end-stage liver disease (ESLD) requiring transplantation. Seven patients (2.2%) developed VAP, of which four did not survive (57.1%), suggesting higher mortality (p-value < 0.001) from VAP. A pre-operative Child-Turcotte-Pugh (CTP) score >13 was associated with higher post-operative mortality (p-value 0.041). The presence of VAP significantly increased intensive care units (ICU) length of stay (p-value 0.018) and mortality (p-value 0.001).

**Conclusion:**

This study showed a low prevalence of VAP in LDLT patients; however, they stayed in the ICU for longer periods and had higher mortality. Considering the complexity and cost of liver transplantation and ICU care, adherence to VAP bundles and purpose-built facilities would prevent VAP and minimize the cost specifically in countries with limited resources.

## INTRODUCTION

Ventilator-associated pneumonia (VAP) is defined, according to Centre for Disease Control (CDC) guidelines, as pneumonia that develops 48-72 hours after endotracheal intubation,^1^-^3^ supported by clinical features, imaging, and cultures from sputum, bronchoalveolar lavage (BAL), or tracheal aspirate. Early-onset VAP is defined as pneumonia that appears within four days following intubation, and late-onset VAP usually develops beyond four days and is more frequently caused by multidrug-resistant (MDR) bacteria.^1^ Male sex, trauma admission, and moderately severe underlying illness are independent risk factors for developing VAP.^2^ The incidence of VAP varies between 5-40% with mortality around 10%,^4^ which can be as high as 25-75%,^5^,^6^ depending on resource availability.

The incidence of VAP in orthotopic liver transplant (OLT) patients is between 5% and 48%, with mortality ranging between 36% and 53%.^7^ Siniscalchi A et al.^8^ reported blood transfusion, mechanical ventilation duration, preoperative hospitalization, and a higher MELD-Na score as significant risk factors for VAP in OLT patients. The aim of our study was to ascertain the prevalence, risk factors, and survival outcomes of post-LDLT patients with VAP in a resource-limited, newly established transplant ICU.

## METHODOLOGY

In this retrospective study, we collected data from 324 patients who underwent LDLT at the Pakistan Kidney and Liver Institute and Research Centre (PKLI-RC) between January 1^st^, 2022 to December 31^st^, 2023.

### Inclusion criteria:

- Patients above 15 years of age who underwent LDLT were enrolled.

### Exclusion criteria:

- Patients who (a) had symptoms or diagnosis of pneumonia prior to intubation and (b) diagnosis of pneumonia within the first 48 hours post-intubation.
- The diagnosis of VAP in our study was confirmed by clinical and radiological findings, along with microbiological isolation of pathogens. Data included demographic information, laboratory findings, cultures from blood/sputum/tracheal aspirate or bronchoalveolar lavage (BAL), BioFire FilmArray Pneumonia Panel plus (BFPP), model for end-stage Liver Disease-Na (MELD-Na), Child-Turcotte-Pugh (CTP) score, Chest X-ray and/or CT scan findings, and duration of ICU stay.

### Ethical Approval:

The PKLI-RC hospital’s ethics committee (PKLI-IRB/AP/157) granted ethical approval, and there are no conflicts of interest to report.

### Statistical Analysis:

The analysis was conducted using the IBM SPSS 27 statistical analysis tool. After checking the distribution of numerical variable using Shapiro wilk test, numerical data such as age, hospital stay, and biochemistry were presented in the form of median and interquartile range (IQR), and categorical data such as gender, risk factors, and diagnosis were presented in the form of frequency and percentages. The Wilcoxon signed-rank sum test was used to determine the association between numerical data.

The association of different categorical variables with survival status was determined using the chi-square test and relevant tests in the case of specific conditions. To determine the difference between survival rates, the log-rank test was applied for overall events and Breslow tests for early events. A p-value <0.05 was considered statistically significant. Crude odds ratios with 95% confidence intervals were determined for chance of death.

## RESULTS

Among 324 LDLT patients, 253 (78.1%) were male and 71 (21.9%) females, with median age of 50 (IQR 43-56) years. Hepatitis C virus (HCV; 192 patients {59.3%}), Hepatitis B virus (HBV; 54 patients {16.7%}), Non-alcoholic steatohepatitis (NASH; 20 patients {6.2%}), Non-B Non-C (24 patients {7.4%}) and Budd-Chiari syndrome (12 patients {3.7%} are the commonly found etiologies of decompensated liver disease (DCLD) followed by other causes including alcoholic cirrhosis (eight patients {2.5%), autoimmune hepatitis (AIH; six patients {0.9%}), primary hyperoxaluria (six patients {0.9%}), primary biliary cirrhosis (PBC; four patients {1.2%}), primary sclerosing cholangitis (two patients {0.6%}) and Wilson disease (one patients {0.3%}). Hepatocellular Carcinoma (HCC) was present in 83 patients (25.6%) mostly secondary to HCV (67 out of 192 HCV patients {34.9%}, followed by HBV (9 out of 54 HBV patients {16.7%}. One patient underwent a liver transplant for acute liver failure secondary to Hepatitis E virus infection. Of the seven VAP patients, five had HCV-related cirrhosis and one each had Non-B Non-C and Budd-Chiari Syndrome. The common comorbidities were diabetes mellitus (18.2%), hypertension (11.1%) and end-stage renal dysfunction (ESRD; 2.16%).

The overall pre-transplant median MELD Na was 18 (IQR 14-22) and CTP was 6 (IQR 7-10). Ventilator associated pneumonia (VAP) was observed in 7 (2.20%) patients with median age of 51.0 (IQR 36-57) years, while median age of non-VAP patients was 50 (IQR 43-56) years, p-value 0.802 (Table-I). Of the seven VAP patients, there were four males and three females. The median length of ICU stay in patients with VAP was 24 days (IQR 8-30) vs 5 days (IQR 4-6) in non-VAP patients (p <0.001).

**Table-I:** Comparison between patients with VAP vs No VAP.

Variable	Stratum	VAP		Total	p-value	Odds (95% C.I.)
		No	Yes			
Median Age (IQR)		50 (43-56)	51 (36-57)	50 (43-56)	0.802	
Median Length of ICU stay (IQR)		5 (4-6)	24 (8-30)	5 (4-6)	0.001*	
Median MELD-Na (IQR)		18 (14-22)	19 (12-26)	18 (14-22)	0.875	
Median CTP (IQR)		9 (7-10)	9 (7-13)	6 (7-10)	0.726	
Gender, n (%)	Male	249 (78.5%)	4 (57.1%)	253 (78.1%)	0.180	
	Female	68 (21.5%)	3 (42.9%)	71 (21.9%)		
Outcome, n (%)	Survived	310 (97.8%)	3(42.9%)	313 (96.6%)	<0.001*	59.05 (95% C.I. 11.07-314.91)
	Expired	7 (2.2%)	4 (57.1%)	11 (3.4%)		
MELD Na, n (%)	≤18	179 (56.5%)	2 (28.6%)	181 (55.9%)	0.248	3.25 (95% C.I. 0.62 - 16.97)
	>18	138 (43.5%)	5 (71.4%)	143 (44.1%)		
MELD Na, n (%)	<21	208 (65.6%)	2 (28.6%)	210 (64.8%)	0.055	4.77 (95% C.I. 0.91-24.99)
	>21	109 (34.4%)	5 (71.4%)	114 (35.2%)		
CTP, n (%)	<13	307 (96.8%)	7 (100%)	314 (96.9%)	1.000	
	>13	10 (3.2%)	0	10 (3.1%)		
Hepatic-hydrothorax, n (%)	No	298 (94.0%)	7 (100%)	305 (94.1%)	1.000	
	Yes	19 (6.0%)	0	19 (5.9%)		

The median MELD-Na score for VAP patients was compared with non-VAP patients (Table-I) which was not statistically significant. Neither MELD-Na score showed any statistically significant difference keeping cut-off ≤/> 18 and ≤/> 21 comparing the groups with and without VAP and in the survival vs. non-survival groups (Table-II). Similarly, no statistically significant difference was found on comparing median Child-Turcotte-Pugh (CTP) scores with cut off ≤ / > 13 between VAP-patients and non-VAP patients (Table-I), However, there was a significant difference in survival (p-value 0.041) between the two groups (Table-II). Odds of death in patients with CTP score more than 13, was 8.47 (95% CI: 1.57 to 45.70) times higher than patients with CTP < 13 which was a statistically significant association between survival and CTP score.

Among the seven patients who developed VAP, four had Klebsiella pneumoniae, three had Escherichia coli, including one co-infection with Klebsiella, while one had Pseudomonas isolated from tracheal aspiration. Of the four Klebsiella pneumoniae patients, one had polymicrobial infections with Acinetobacter and Burkholderia cepacia, and another had E. Coli; both patients died. Among the other two deaths, one had Klebsiella pneumonia with secondary bacteremia, and the second had Pseudomonas in tracheal aspirate culture complicated with Enterobacter bacteremia. Out of 324 patients, 19 had hepatic hydrothorax (5.9%), but none of them developed VAP, and none of them died.

The overall mortality was 3.4% (11 out of 324 LDLT patients), including four deaths from VAP, while remaining seven deaths had septic shock secondary to extra-pulmonary source of infections. The 30-day mortality in non-VAP was 2.2% (seven deaths out of 317) and in VAP patients was 57.1% (four out of seven patients) (Table-II), suggesting higher mortality from VAP (p< 0.001). Odds of death in VAP was 59.05 (95% C.I.11.07- 314.91) times higher than non-VAP. The association between VAP status and survival was statistically significant (p <0.001) (Table-II).

**Table-II:** Outcome of patients.

Variable	Stratum	Outcome		Total	p-value	Odds (95% C.I.)
		Expired	Survived			
Median Age (IQR)		36 (31-55)	50 (43-56)	50 (43-56)	0.102	
Median Length of ICU Stay (IQR)		8 (4-22)	5 (4-6)	5 (4-6)	0.144	
Median MELD-Na (IQR)		21 (9-25)	18 (14-22)	18 (14-22)	0.604	
Median CTP score (IQR)		9 (7-12)	9 (7-10)	6 (7-10)	0.352	
Gender, n (%)	Male	7 (63.6%)	246 (78.6%)	253 (78.1%)	0.265	
	Female	4 (36.4%)	67 (21.4%)	71 (21.9%)		
MELD Na, n (%)	≤18	5 (45.5%)	176 (56.2%)	181 (55.9%)	0.546	1.54(0.46-5.16) ^ô^
	>18	6 (54.5%)	137 (43.8%)	143 (44.1%)		
MELD Na, n (%)	<21	5 (45.5%)	205 (65.5%)	210 (64.8%)	0.204	2.28(0.68-7.63) ^ô^
	>21	6 (54.5%)	108 (34.5%)	114 (35.2%)		
CTP, n (%)	≤13	9 (81.8%)	305 (97.4%)	314 (96.9%)	0.041*	8.47(1.57-45.70) ^ô^
	>13	2 (18.2%)	8 (2.6%)	10 (3.1%)		
Hepatic hydrothorax, n (%)	No	11 (100%)	294 (93.9%)	305 (94.1%)	1.000
	Yes	0	19 (6.1%)	19 (5.9%)	
VAP, n (%)	Yes	4 (36.4%)	3 (1.0%)	7 (2.2%)	<0.001*	59.05(11.07-314.90)
	No	7 (63.6%)	310 (99.0%)	317 (97.8%)		

The ô odds were computed for raised values.

**Fig.1:**
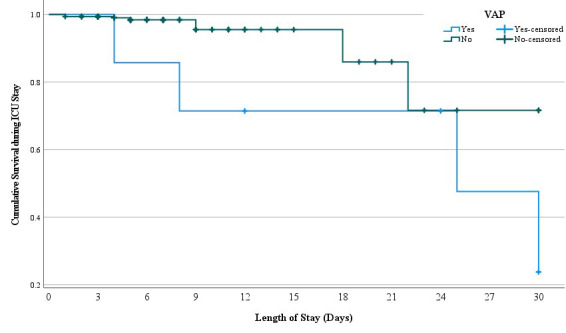
Kaplan-Meier curve showing the comparative survival of VAP and non-VAP patients.

The comparative survival between VAP and non-VAP patients is shown in the Kaplan-Meier curve (Fig.1). The estimated probability of survival on day seven in VAP patients was 85.7%, whereas in non-VAP was 98.4%, while on 14^th^ day for VAP patients it was 71.4%, for non-VAP patients it was 98.4%, and on 30^th^ day it was 23.8% vs. 71.6%, respectively. Even though the survival rates of patients with and without VAP differed significantly (p< 0.001), we could not find a significant difference in terms of the probability of survival over time (p-value 0.062).

## DISCUSSION

This study found low prevalence of VAP in our LDLT patients (2.2%) as compared to other published data (5% to 48%) in orthotopic liver transplant (OLT) patients,^7^,^8^ The estimated incidence of VAP varies from 5% to 40%^4^,^3^ among ICU patients, based on the criteria, screening methods, and patient demographics.

The causes of chronic liver disease (CLD) in developed countries differ from those in developing countries. According to GBD Study 2017, the global burden of CLD is about 1.5 billion persons, mostly secondary to non-alcoholic fatty liver disease (NAFLD; 60%), HBV (29%), HCV (9%), and alcohol-related liver disease (ALD; 2%).^9^ While in Pakistan, HCV is the leading cause of CLD, with a prevalence of 5% and an infection rate of 11.55% in adult population.^10^ HCV infection was the most common cause of ESLD and HCC, leading to transplant in our patients, followed by HBV infection, non-B non-C, and HCC, while diabetes was the most common comorbidity; however, none of the etiologies, age, sex, or comorbidities were linked to the development of VAP in our study.

Post liver transplant patients, especially during first month, are at high risk of bacterial infections ranging from 20% to 80%, carrying higher mortality.^11^ In this study, the overall mortality was low at 3.4% (11 patients), with septic shock being the leading cause of death. Gram-positive bacteria, such as Staphylococcus aureus, and gram-negative bacteria, such as Pseudomonas aeruginosa, Klebsiella pneumoniae, and Escherichia coli are the most frequent causes of VAP.^12^ The most prevalent organism in our study was Klebsiella pneumoniae, which had a significant mortality rate; three out of four patients, including those with polymicrobial infections, died from the disease. In a study of 70 patients,^12^ 43 cases (61.4 %) developed VAP, mostly secondary to HV-positive K. pneumoniae with a higher mortality rate than HV-negative strains, although antibiotic resistance was more frequent in HV- negative strains than in HV-infected patients.

Ventilator-associated pneumonia (VAP) is commonly recognized as the source of infections, specifically in transplant recipients, and has high mortality rate ranging between 24% and 50%, which can be as high as 76%, especially in cases secondary to high-risk pathogens.^12^,^13^ In our study also, VAP has shown to be the major cause of death, as four out of seven (57.1%) patients with VAP did not survive, this is higher than previous studies.^4^,^7^ Our study also confirmed the statistically significant impact on ICU length of stay (p-value 0.001), as VAP increased the ICU length of stay from five days to 24 days; the higher the length of stay, the greater the mortality (Fig.1), which is consistent with previous reports.^7^

The MELD score and CTP scores are the two scores typically used to determine mortality risk and the need for transplant. According to a liver transplant study, patients who underwent LDLT for HCC and had a pre-transplant MELD score of > 20 were at a higher risk of post-LT septic shock and death.^14^ Another study suggested that recipients with high MELD scores are less tolerant of marginal grafts.^15^ However, there are also reports suggesting no effect on outcomes from high pre-liver transplant (LT) MELD scores following LDLT.^16^,^17^ We used the MELD-Na score instead of MELD, as MELD-Na is known to identify patients at a higher mortality risk^18^ and predict mortality better than the MELD Score.^19^

However, in this study, although the MELD-Na score was higher in patients who developed VAP and in patients who did not survive, it was not statistically significant. In contrast to the MELD-Na score, we found that patients with CTP < 13 had better chances of survival than those with CTP >13. Thus, our study suggests that future prospective studies are required to include CTP score as an important variable where CTP score can help in screening patients for transplant. In addition, our study did not find any statistical difference in pre-operative MELD-Na scores between patients with and without VAP, or between survivors and non-survivors. We do not perform routine gastric residual volume (GRV) measurements as part of VAP bundle as it is no more considered a risk factor for VAP.^20^ The low prevalence of VAP in our ICU can be attributed to strict infection control policies including adherence to the VAP prevention bundle protocol. However, once it develops, it results in a longer stay in ICU and a higher death rate.

### Strength of the study:

The strength of this study was the diagnostic accuracy. We strictly followed the diagnostic criteria for VAP supplemented by reliable culture reports from tracheal aspirate, BAL and used BioFire FilmArray Pneumonia Panel plus (BFPP) when needed, which avoided the risk of bias. We found Klebsiella pneumoniae as the most common organism but as the sample size was small, therefore we suggest further large-scale studies to determine the prevalence of Klebsiella in post LDLT patients. However, once there is VAP, the treatment regimen should cover Klebsiella pneumoniae as failure to do so carries high mortality.

### Limitations:

First, as this was a retrospective study, a specific protocol was not established. Second, laboratory data, including CTP and MELD-Na scores, were calculated preoperatively when the patient was placed on the surgical waiting list, instead of the average from perioperative periods, resulting in bias and a lack of dynamic assessment. Third, a type II error may have existed because of the relatively small sample size.

## CONCLUSION

Our study reports the low prevalence of VAP in LDLT patients which is due to the strict adherence to VAP bundle in our ICU; however, once developed, it raises mortality and length of stay in ICU. Patients with a CTP score <13 have much better survival than those to >13. Thus, future studies should also consider high pre-transplant CTP scores as a risk factor for recipients. Liver transplant and ICU care are costly, adherence to the VAP bundle, and staff training would be cost-effective and improve survival, especially in countries with limited resources. We suggest transplant centers strictly follow the clinical guidelines for the prevention of hospital acquired infections including VAP that directly link to survival of patients.

## References

[1] Liu Y, Di Y, Fu S (2017). Risk factors for ventilator-associated pneumonia among patients undergoing major oncological surgery for head and neck cancer. Front Med.

[2] American Thoracic Society, Infectious Diseases Society of America (2005). Guidelines for the management of adults with hospital-acquired, ventilator-associated, and healthcare-associated pneumonia. Am J Respir Crit Care Med.

[3] Johnstone J, Muscedere J, Dionne J, Duan E, Rochwerg B, Centofanti J (2023). Definitions, rates and associated mortality of ICU-acquired pneumonia: A multicenter cohort study. Prevention of Severe Pneumonia and Endotracheal Colonization Trial (PROSPECT) Investigators and the Canadian Critical Care Trials Group. J Crit Care.

[4] Papazian L, Klompas M, Luyt CE (2020). Ventilator-associated pneumonia in adults: a narrative review. Intensive Care Med.

[5] Charles MP, Kali A, Easow JM, Joseph NM, Ravishankar M, Srinivasan S (2014). Ventilator-associated pneumonia. Australas Med J.

[6] Harsha VP, Virendra CP (2017). Incidence, bacteriology, and clinical outcome of ventilator associated pneumonia at tertiary care hospital. J Nat Sci Biol Med.

[7] Pellegrino CM, Codeluppi M, Assenza S, Cocchi S, Di Benedetto F, Girardis M (2008). Incidence and clinical outcomes of ventilator associated pneumonia in liver transplant and non-liver transplant surgical patients. Transplant Proc.

[8] Siniscalchi A, Aurini L, Benini B, Gamberini L, Nava S, Viale P (2016). Ventilator associated pneumonia following liver transplantation: Etiology, risk factors and outcome. World J Transplant.

[9] James SL, Abate D, Abate KH, Abay SM, Abbafati C, Abbasi N (2018). GBD 2017 Disease and Injury Incidence and Prevalence Collaborators. Global, regional, and national incidence, prevalence, and years lived with disability for 354 diseases and injuries for 195 countries and territories, 1990-2017: a systematic analysis for the Global Burden of Disease Study 2017. Lancet.

[10] Arshad A, Ashfaq UA (2017). Epidemiology of Hepatitis C Infection in Pakistan: Current Estimate and Major Risk Factors. Crit Rev Eukaryot Gene Expr.

[11] Fagiuoli S, Colli A, Bruno R, Craxì A, Gaeta GB, Grossi P (2014). Management of infections pre- and post-liver transplantation: report of an AISF consensus conference. J Hepatol.

[12] Guo S, Xu J, Wei Y, Xu J, Li Y, Xue R (2016). Clinical and molecular characteristics of Klebsiella pneumoniae ventilator-associated pneumonia in mainland China. BMC Infect Dis.

[13] Chastre J, Fagon JY (2002). Ventilator-associated pneumonia. Am J Respir Crit Care Med.

[14] Hung HC, Lee JC, Wang YC, Cheng CH, Wu TH, Wu TJ (2022). Living-Donor Liver Transplantation for Hepatocellular Carcinoma: Impact of the MELD Score and Predictive Value of NLR on Survival. Curr Oncol.

[15] Hoffmann K, Hinz U, Hillebrand N, Radeleff BA, Ganten TM, Schirmacher P (2011). Risk factors of survival after liver transplantation for HCC: A multivariate single-center analysis. Clin Transplant.

[16] Yadav SK, Saraf N, Saigal S, Choudhary NS, Goja S, Rastogi A (2017). High MELD score does not adversely affect outcome of living donor liver transplantation: Experience in 1000 recipients. Clin Transplant.

[17] Klein KB, Stafinski TD, Menon D (2013). Predicting Survival after Liver Transplantation Based on Pre-Transplant MELD Score: A Systematic Review of the Literature. PLoS ONE.

[18] Brown C, Aksan N, Muir AJ (2022). MELD-Na Accurately Predicts 6-Month Mortality in Patients with Decompensated Cirrhosis: Potential Trigger for Hospice Referral. J Clin Gastroenterol.

[19] Kim WR, Biggins SW, Kremers WK, Wiesner RH, Kamath PS, Benson JT (2008). Hyponatremia and mortality among patients on the liver-transplant waiting list. N Engl J Med.

[20] Faramarzi E, Mahmoodpoor A, Hamishehkar H, Shadvar K, Iranpour A, Sabzevari T (2020). Effect of gastric residual volume monitoring on incidence of ventilator-associated pneumonia in mechanically ventilated patients admitted to intensive care unit. Pak J Med Sci.

